# Tocilizumab versus anakinra in COVID-19: results from propensity score matching

**DOI:** 10.3389/fimmu.2023.1185716

**Published:** 2023-05-26

**Authors:** Robin Arcani, Florian Correard, Pierre Suchon, Gilles Kaplanski, Rodolphe Jean, Raphael Cauchois, Marine Leprince, Vincent Arcani, Julie Seguier, Benjamin De Sainte Marie, Baptiste Andre, Marie Koubi, Pascal Rossi, Stéphane Gayet, Nirvina Gobin, Victoria Garrido, Joris Weiland, Elisabeth Jouve, Anne-Laure Couderc, Patrick Villani, Aurélie Daumas

**Affiliations:** ^1^ Internal Medicine and Therapeutics Department, CHU La Timone, Assistance Publique-Hôpitaux de Marseille (AP-HM), Marseille, France; ^2^ Center for Cardiovascular and Nutrition Research (C2VN), INRA 1260, INSERM UMR_S 1263, Aix-Marseille University, Marseille, France; ^3^ Pharmacy Department, CHU La Timone, AP-HM, Marseille, France; ^4^ Hematology Laboratory, CHU La Timone, Assistance Publique-Hôpitaux de Marseille (AP-HM), Marseille, France; ^5^ Internal Medicine and Clinical Immunology Department, CHU La Conception, Assistance Publique-Hôpitaux de Marseille (AP-HM), Marseille, France; ^6^ Internal Medicine Department, CHU La Timone, Assistance Publique-Hôpitaux de Marseille (AP-HM), Marseille, France; ^7^ Department of Internal Medicine, CHU Nord, Assistance Publique-Hôpitaux de Marseille (AP-HM), Marseille, France; ^8^ Service Evaluation Médicale, CHU la Conception, Assistance Publique-Hôpitaux de Marseille (AP-HM), Marseille, France; ^9^ Internal Medicine, Geriatrics and Therapeutic Department, CHU Sainte-Marguerite, Assistance Publique-Hôpitaux de Marseille (AP-HM), Marseille, France; ^10^ Aix-Marseille University, CNRS, EFS, ADES, Marseille, France

**Keywords:** anakinra, tocilizumab, anti-interleukin drugs, COVID-19, mortality, SARS-CoV-2, anti-interleukin 6, interleukin 1 receptor antagonist

## Abstract

**Background:**

Tocilizumab and anakinra are anti-interleukin drugs to treat severe coronavirus disease 2019 (COVID-19) refractory to corticosteroids. However, no studies compared the efficacy of tocilizumab versus anakinra to guide the choice of the therapy in clinical practice. We aimed to compare the outcomes of COVID-19 patients treated with tocilizumab or anakinra.

**Methods:**

Our retrospective study was conducted in three French university hospitals between February 2021 and February 2022 and included all the consecutive hospitalized patients with a laboratory-confirmed severe acute respiratory syndrome coronavirus 2 (SARS-CoV-2) infection assessed by RT-PCR who were treated with tocilizumab or anakinra. A propensity score matching was performed to minimize confounding effects due to the non-random allocation.

**Results:**

Among 235 patients (mean age, 72 years; 60.9% of male patients), the 28-day mortality (29.4% *vs.* 31.2%, p = 0.76), the in-hospital mortality (31.7% *vs.* 33.0%, p = 0.83), the high-flow oxygen requirement (17.5% *vs.* 18.3%, p = 0.86), the intensive care unit admission rate (30.8% *vs.* 22.2%, p = 0.30), and the mechanical ventilation rate (15.4% *vs.* 11.1%, p = 0.50) were similar in patients receiving tocilizumab and those receiving anakinra. After propensity score matching, the 28-day mortality (29.1% *vs.* 30.4%, p = 1) and the rate of high-flow oxygen requirement (10.1% *vs.* 21.5%, p = 0.081) did not differ between patients receiving tocilizumab or anakinra. Secondary infection rates were similar between the tocilizumab and anakinra groups (6.3% *vs.* 9.2%, p = 0.44).

**Conclusion:**

Our study showed comparable efficacy and safety profiles of tocilizumab and anakinra to treat severe COVID-19.

## 1 Introduction

Coronavirus disease 2019 (COVID-19) is caused by severe acute respiratory syndrome coronavirus 2 (SARS-CoV-2) (1) and has led to more than 6 million deaths around the world (2). COVID-19 can range from a simple asymptomatic viral infection to acute respiratory distress syndrome (ARDS) requiring intensive care; 15%–20% of hospitalized patients develop severe pneumonia (3, 4). Currently, the standard of care for in-hospitalized patients requiring oxygen is based on corticosteroids (5, 6), which have broad-spectrum anti-inflammatory actions. Despite the extensive use of dexamethasone, 15%–30% of patients remain refractory to corticosteroids and require intubation or progress to death (5–7).

Tocilizumab (an interleukin (IL)-6 receptor antagonist) and anakinra (an IL-1 receptor antagonist) have been proven effective in reducing mortality in patients with severe inflammatory COVID-19 (8–12). In France, since 2021, the French High Council for Public Health (HCSP) recommends adding anti-IL-6 or anti-IL-1 drugs in patients requiring high-flow oxygen therapy and having a marked inflammatory state in the absence of improvement (C-reactive protein (CRP) >75 mg/L) after 48 h of the standard of care, including dexamethasone (13). However, to date, there is no evidence showing which anti-IL drug is the most effective. Scarce data have compared the efficacy of tocilizumab versus anakinra in dexamethasone-refractory COVID-19 patients (14, 15). We aimed to retrospectively compare the outcomes of dexamethasone-refractory COVID-19 patients treated with tocilizumab or anakinra using propensity score matching.

## 2 Materials and methods

### 2.1 Patients

We retrospectively included all consecutive adult patients (aged ≥18 years) admitted with a laboratory-confirmed SARS-CoV-2 infection assessed by RT-PCR on nasopharyngeal swabs requiring oxygen, who were dexamethasone-refractory (defined by an absence of clinical improvement and/or an absence of a decrease in CRP after 72 h of dexamethasone) and treated with tocilizumab or anakinra between February 2021 and February 2022 in six internal medicine departments in three hospitals (La Timone, La Conception and Hôpital Nord, and University Hospitals of Marseille, France). Patients were not included if they had received both anti-IL drugs or one anti-IL and one JAK inhibitor to treat COVID-19 or if there were insufficient patient follow-up data to perform analysis (e.g., patient transferred to another hospital).

Clinical, biological, radiological, and follow-up data were collected from electronic medical records. Patients were divided into three groups based on low-dose chest computed tomography (CT) extent of lung parenchymal lesions (minimal, <10%; moderate, 10%–25%; and severe, >25%). The vaccine status of the patients was unvaccinated, partially vaccinated, or fully vaccinated (0, 1, or 2 doses received, respectively).

Patients received tocilizumab (8 mg/kg, 800 mg maximum) intravenously over a 1-h infusion once or repeated after 24 h, according to the opinion of the attending clinician. Patients were treated with subcutaneous anakinra 100 mg/day until clinical improvement (maximum 10 days) or with 300 mg/day for 5 days and then tapering 200 mg/day for 2 days and 100 mg/day for 1 day. All patients were treated with dexamethasone (6 mg/day, administered intravenously, until oxygen discontinuation). Patients could also have received anti-spike monoclonal antibodies [casirivimab/imdevimab (Ronapreve^®^), or sotrovimab (Xevudy^®^) according to the SARS-CoV-2 variants], antibiotics, and prophylactic (enoxaparin 40 mg/day), intermediate (enoxaparin 40 mg twice a day), or therapeutic (tinzaparin 175 anti-Xa IU/kg/day) thromboprophylaxis according to the current recommendations from the French Society of Critical Care (16) until discharge.

Patients were considered under immunosuppressive therapy when they took anti-rejection therapy, immunosuppressive therapy, corticosteroids > 10 mg/day, biotherapy, or chemotherapy before COVID-19. Patients were considered with a secondary infection when they needed a new antibiotic 48 h after the introduction of the anti-IL drug or when a new bacterial infection was identified (by culture or molecular testing).

### 2.2 Ethics

This study was approved by the Institutional Review Board of Assistance Publique - Hôpitaux de Marseille (GDPR number PADS22-339). The study was conducted in compliance with good clinical practices and the Declaration of Helsinki principles. Formal approval from an ethics committee was not required for this observational study.

### 2.3 Statistical analysis

Quantitative variables were described using means and standard deviations (SD); categorical variables were described using numbers and percentages. Quantitative data were compared using Student’s t-test or the Mann–Whitney U test, while qualitative data were compared with the chi-square or Fisher’s exact test when appropriate. A propensity score matching was performed to minimize confounding effects due to the non-random allocation. The propensity score matching function matched the two groups of patients with an anti-IL drug (tocilizumab or anakinra) as the dependent variable. Based on risk factors described in the literature (17–21), propensity score matching of 1:1 was performed with age, sex, vaccine status (partial/full versus none), the extent of lung involvement, comorbidities (hypertension, cancer, chronic kidney disease, diabetes, and obesity), a high-flow requirement before anti-IL introduction, CRP level at the anti-IL drug introduction, use of anti-spike monoclonal antibodies, and thromboprophylaxis as covariates using the optimal method. To confirm the results found with the optimal method, we performed the nearest neighbor propensity score matching method (caliper = 0.25). After matching, McNemar’s test was used to test the association of the mortality rate/rate of high-flow oxygen requirement with the anti-IL drug between matched pairs. The tests were two-sided. p-Values <0.05 were considered significant. All analyses were performed with R software (R Foundation for Statistical Computing, Vienna, Austria), and the propensity score was performed with the MatchIt package.

## 3 Results

### 3.1 Characteristics of the population

During the study period, 259 patients were treated with anti-IL drugs; 19 patients were excluded because they received tocilizumab and anakinra (n = 16) or JAK inhibitor and anakinra (n = 3), and five patients were excluded because of a lack of follow-up data. A total of 235 patients were included in the analysis. The main characteristics of the population are presented in **Table 1**. The mean age was 72 ± 15 years (range, 28–99), with 143 male patients (60.9%). The main comorbidities were hypertension (51.5%), type 2 diabetes (34.0%), obesity (23.4%), overweight (19.4%), chronic kidney disease (18.7%), coronary artery disease (14.0%), pulmonary diseases (chronic obstructive pulmonary disease, asthma, or obstructive sleep apnea, 13.6%), and active cancer (13.2%). Of the total patients, 21 (8.9%) received previous immunosuppressive therapy, and 116 (49.4%) had a “not to be resuscitated” status.

**Table 1:** Description of the cohort.

Characteristics	Tocilizumab group (n = 126)	Anakinra group (n = 109)	Overall population (n = 235)	p-Value^a^
Age^b^	73 ± 15 (32–98)	72 ± 16 (28–99)	72 ± 15 (28–99)	0.71
Male gender	76 (60.3)	67 (61.5)	143 (60.9)	0.86
Length of hospitalization^b^	13 ± 10 (1–89)	13 ± 8 (4–44)	13 ± 9 (1–89)	0.95
Vaccinated - Partially - Fully	28 (22.2) 9 (7.1) 19 (15.1)	33/103 (32.0) 3/103 (2.9) 30/103 (29.1)	61/229 (26.6) 12/229 (5.2) 49/229 (21.4)	0.095 0.15 **0.0099**
Body mass index (kg/m^2^) ^b^	28 ± 7 (16–57)	26 ± 6 (16–43)	27 ± 6 (16–57)	0.20
Extent of lung involvement - No COVID-19 lesion - <10% - 10%–25% - >25%	4/119 (3.4) 16/119 (13.4) 50/119 (42.0) 49/119 (41.2)	4/101 (4.0) 10/101 (9.9) 26/101 (25.7) 61/101 (60.4)	8/220 (3.6) 26/220 (11.8) 76/220 (34.5) 110/220 (50)	0.81 0.42 **0.011** **0.0045**
SARS-CoV-2 variant - Alpha variant - Beta variant - Delta variant - Omicron variant	22/46 (47.8) 2/46 (4.3) 21/46 (45.7) 1/46 (2.2)	3/65 (4.6) 3/65 (4.6) 30/65 (46.2) 29/65 (44.6)	25/111 (22.5) 5/111 (4.5) 51/111 (45.9) 30/111 (27.0)	**<0.001** 0.95 0.96 **<0.001**
Comorbidities - Hypertension - Coronaropathy disease - Stroke - Venous thromboembolism - Pulmonary diseases - Cancer - Chronic kidney disease - Diabetes - Obesity - Overweight - Immunosuppressive therapy	62 (49.2) 13 (10.3) 2 (1.6) 2 (1.6) 12 (9.5) 18 (14.3) 15 (11.9) 36 (28.6) 26/116 (22.4) 23/116 (19.8) 5 (4.0)	59 (49.6) 20 (18.3) 7 (6.4) 2 (1.8) 20 (18.3) 13 (11.9) 29 (24.4) 44 (40.4) 21/85 (24.7) 16/85 (18.8) 16 (14.7)	121 (51.5) 33 (14.0) 9 (3.8) 4 (1.7) 32 (13.6) 31 (13.2) 44 (18.7) 80 (34.0) 47/201 (23.4) 39/201 (19.4) 21 (8.9)	0.45 0.077 0.054 0.88 **0.049** 0.59 **0.0040** 0.057 0.70 0.86 **0.0041**
High-flow oxygen introduction before anti-interleukin introduction	104 (82.5)	46 (42.2)	150 (63.8)	**<0.001**
Ferritin (µg/L) at anti-interleukin introduction^b^	1,393 ± 1,225 (118–6,607)	1,571 ± 1,584 (96–10,555)	1,496 ± 1,442 (96–10,555)	0.42
CRP (mg/L) at anti-interleukin introduction^b^	119 ± 76 (8–349)	129 ± 79 (17–351)	123 ± 77 (8–351)	0.30
Associated therapies - Anti-spike monoclonal antibody - Any antibiotics - Third-generation cephalosporin - Azithromycin - Piperacillin–tazobactam - Carbapenem - Prophylactic thromboprophylaxis - Intermediate thromboprophylaxis - Therapeutic thromboprophylaxis	0 (0) 56 (44.4) 41 (32.5) 15 (11.9) 17 (13.5) 0 (0) 57 (45.2) 30 (23.8) 37 (29.4)	22 (20.2) 96 (88.1) 76 (69.7) 68 (62.4) 39 (35.8) 11 (10.1) 28 (25.7) 35 (32.1) 44 (40.4)	22 (9.4) 152 (64.7) 117 (49.8) 83 (35.3) 56 (23.8) 11 (4.7) 85 (36.2) 65 (27.7) 81 (34.5)	**<0.001** **<0.001** **<0.001** **<0.001** **<0.001** **<0.001** **0.0019** 0.16 0.077

Note. p-Value <0.05 in bold.

CRP, C-reactive protein.

a Comparison between tocilizumab and anakinra groups.

b Mean ± standard deviation (range).

Vaccine status was available for 229 patients: 61 patients (26.6%) received at least one dose of vaccine, 12 patients (5.2%) were partially vaccinated (only one dose of vaccine), and 49 (21.4%) were fully vaccinated (two doses of vaccine). Assessment of SARS-CoV-2 variants was available in 111 patients: 25 (22.5%) carried the alpha variant, 5 (4.5%) carried the beta variant, 51 (45.9%) carried the delta variant, and 30 (27.0%) carried the omicron variant. Lung CT showed typical lesions of COVID-19 in 212 out of 220 patients (96.4%), consisting of minimal, moderate, and severe lesions in 11.8%, 34.5%, and 50.0%, respectively. Of 152 patients (64.7%) who received antibiotics, 117 of them (49.8%) received third-generation cephalosporin, 83 patients (35.3%) received azithromycin, 56 patients (23.8%) received piperacillin–tazobactam, and 11 patients (4.7%) received carbapenem. Sixty-six patients (28.1%) received only one antibiotic, 61 patients (26.0%) received two antibiotics, 21 patients (8.9%) received three antibiotics, and four patients (1.7%) received four antibiotics. Of 235 patients, 231 (98.3%) were treated with low-molecular-weight heparin at prophylactic, intermediate, or therapeutic doses of 36.2%, 27.7%, and 34.5%, respectively; 22 patients (9.4%) were treated with monoclonal anti-spike antibodies; 150 patients (63.8%) were treated with an anti-IL drug once under high-flow oxygen. At the time of anti-IL introduction, ferritin was 1,496 ± 1,442 µg/L (range, 96–10,555), and C-reactive protein was 123 ± 77 mg/L (range, 8–351).

### 3.2 Comparisons of the baseline characteristics between patients receiving tocilizumab and anakinra

A total of 126 (53.6%) patients received tocilizumab, and 109 (46.4%) patients received anakinra (**Table 1**). The two cohorts were similar in age, gender, and main comorbidities (hypertension, coronaropathy disease, obesity, overweight, and cancer). There were some differences between the two cohorts in terms of vaccine status (fully vaccinated patients: 15.1% of the tocilizumab group *vs.* 29.1% of the anakinra group, p = 0.01), extent of lung involvement (42.0% of intermediate lesions in the tocilizumab group *vs.* 25.7% in the anakinra group, p = 0.011; 41.2% of severe lesions in the tocilizumab group *vs.* 60.4% in the anakinra group, p = 0.0045), SARS-CoV-2 variants (47.8% of alpha variant in the tocilizumab group *vs.* 4.6% in the anakinra group, p < 0.001; 2.2% of omicron variant in the tocilizumab group *vs.* 44.6% in the anakinra group, p < 0.001), comorbidities (9.5% of the tocilizumab group had pulmonary diseases *vs.* 18.3% of the anakinra group, p = 0.049; 11.9% of the tocilizumab group had chronic kidney disease *vs.* 24.4% of the anakinra group, p = 0.0040; 4.0% of the tocilizumab group were under immunosuppressive therapy *vs.* 14.7% of the anakinra group, p = 0.0041), severity of clinical state (82.5% of the tocilizumab group were under high-flow oxygen before anti-IL introduction *vs.* 42.2% of the anakinra group, p < 0.001), thromboprophylaxis management (45.2% of the tocilizumab group received prophylactic dose of low-molecular-weight heparin *vs.* 25.7% of the anakinra group, p = 0.0019), or associated therapies (0% of the tocilizumab group received anti-spike monoclonal antibody *vs.* 20.2% of the anakinra group, p < 0.001; 44.4% of the tocilizumab group received antibiotics *vs.* 88.1% of the anakinra group, p < 0.001).

After propensity score matching using the optimal method (**Table 2**), 79 patients treated with tocilizumab and 79 patients treated with anakinra had similar characteristics (except for anti-spike monoclonal antibodies; no patients in the tocilizumab group received anti-spike monoclonal antibodies *vs.* 26.6% of the anakinra group, p < 0.001; for high-flow oxygen introduction before anti-IL introduction, 73.4% were under high-flow oxygen at tocilizumab introduction *vs.* 43.0% at anakinra introduction). Because of the imbalance of covariates between the two groups, we performed a propensity score matching using the nearest neighbor method (**Table 3**). Similar characteristics were found in 36 patients treated with tocilizumab and 36 patients treated with anakinra.

**Table 2:** Patient characteristics after matching (optimal method).

Characteristics	Tocilizumab group (n = 79)	Anakinra group (n = 79)	p-Value^a^
Age^b^	74 (15)	70 (16)	0.11
Male gender	44 (55.7)	51 (64.6)	0.33
Vaccinated (partially or fully)	22 (27.8)	23 (29.1)	1
Extent of lung involvement - No COVID-19 lesion - <10% - 10%–25% - >25%	5 (6.3) 10 (12.7) 25 (31.6) 35 (44.3)	5 (6.3) 8 (10.1) 19 (24.1) 44 (55.7)	0.70
Hypertension	42 (53.2)	39 (49.4)	0.7
Cancer	10 (12.7)	8 (10.1)	0.80
Chronic kidney disease	11 (13.9)	18 (22.8)	0.22
Diabetes	23 (29.1)	29 (36.7)	0.40
Obesity	14 (17.7)	21 (26.6)	0.25
CRP (mg/L) at anti-interleukin introduction^b^	125 (82)	130 (80)	0.70
Anti-spike monoclonal antibody	0 (0)	21 (26.6)	**<0.001**
Associated anticoagulant: - No anticoagulant - Prophylactic thromboprophylaxis - Intermediate thromboprophylaxis - Therapeutic thromboprophylaxis	2 (2.5) 24 (30.4) 26 (32.9) 27 (34.2)	1 (1.3) 21 (26.6) 29 (36.7) 28 (35.4)	0.87
High-flow oxygen introduction before anti-interleukin introduction	58 (73.4)	34 (43.0)	**<0.001**

Note. p-Value <0.05 in bold.

CRP, C-reactive protein.

a Comparison between tocilizumab and anakinra groups.

b Mean ± standard deviation (range).

**Table 3:** Patient characteristics after matching (nearest neighbor method).

Characteristics	Tocilizumab group (n = 36)	Anakinra group (n = 36)	p-Value^a^
Age^b^	74 (16)	69 (17)	0.25
Male gender	19 (52.8)	20 (55.6)	1
Vaccinated (partially or fully)	14 (38.9)	10 (27.8)	0.45
Extent of lung involvement - No COVID-19 lesion - <10% - 10%–25% - >25%	2 (5.6) 5 (13.9) 8 (22.2) 18 (50)	1 (2.8) 4 (11.1) 13 (36.1) 16 (44.4)	0.74
Hypertension	21 (58.3)	16 (44.4)	0.35
Cancer	4 (11.1)	3 (8.3)	1
Chronic kidney disease	7 (19.4)	5 (13.9)	0.75
Diabetes	9 (25)	9 (25)	1
Obesity	8 (22.2)	9 (25)	1
CRP (mg/L) at anti-interleukin introduction^b^	136 (79)	119 (75)	0.41
Monoclonal anti-spike antibody	0 (0)	0 (0)	
Associated anticoagulant: - No anticoagulant - Prophylactic thromboprophylaxis - Intermediate thromboprophylaxis - Therapeutic thromboprophylaxis	1 (2.8) 14 (38.9) 11 (30.6) 10 (27.8)	1 (2.8) 12 (33.3) 14 (38.9) 9 (25)	0.90
High-flow oxygen introduction before anti-interleukin introduction	12 (33.3)	14 (38.9)	0.81

Note. CRP, C-reactive protein.

a Comparison between tocilizumab and anakinra groups.

b Mean ± standard deviation (range).

### 3.3 Outcomes of patients under anti-IL drugs

The 28-day mortality was similar between the two groups (29.4% in the tocilizumab group *vs.* 31.2% in the anakinra group, p = 0.76), as was in-hospital mortality (31.7% in the tocilizumab group *vs.* 33.0% in the anakinra group, p = 0.83). High-flow oxygen was required in similar proportions (17.5% in the tocilizumab group *vs.* 18.3% in the anakinra group, p = 0.86). Among patients without a “not to be resuscitated” status (n = 119), the transfer rate into intensive care units (ICUs) was similar between the two groups (30.8% in the tocilizumab group *vs.* 22.2% in the anakinra group, p = 0.30). The secondary infections following anti-IL drugs were quite scarce (6.3% in the tocilizumab group *vs.* 9.2% in the anakinra group, p = 0.44) (**Table 4**).

**Table 4:** Outcomes of the cohort.

Outcome	Tocilizumab group (n = 126)	Anakinra group (n = 109)	Overall population (n = 235)	p-Value^a^
Secondary infection	8 (6.3)	10 (9.2)	16 (6.8)	0.44
High-flow oxygen	22 (17.5)	20 (18.3)	42 (17.9)	0.86
Intensive care unit transfer	20/65 (30.8)	12/54 (22.2)	32/119 (26.9)	0.30
Mechanical ventilation	10/65 (15.4)	6/54 (11.1)	16/119 (13.4)	0.50
28-day mortality	37 (29.4)	34 (31.2)	71 (30.2)	0.76
In-hospital mortality	40 (31.7)	36 (33.0)	76 (32.2)	0.83

a Comparison between tocilizumab and anakinra groups.

After propensity score matching using the optimal method, the 28-day mortality (29.1% in the tocilizumab group *vs.* 30.4% in the anakinra group, p = 1) and the rate of high-flow oxygen requirement (10.1% in the tocilizumab group *vs.* 21.5% in the anakinra group, p = 0.081) did not differ between the two groups. Using the nearest neighbor method, we found a similar 28-day mortality (33.3% in the tocilizumab group *vs.* 30.6% in the anakinra group, p = 1) and a similar rate of high-flow oxygen requirement (5.6% in the tocilizumab group *vs.* 13.9% in the anakinra group, p = 0.43) between the pseudo-populations.

## 4 Discussion

We reported a cohort of hospitalized COVID-19 before ICU admission and intubation who were treated with an anti-IL drug. We showed that the 28-day mortality and the high-flow oxygen requirement did not differ according to the treatment received, tocilizumab or anakinra. The two treatments had the same risk of infections.

These results follow the few data available about this subject. IL-1 and IL-6 are two cytokines mainly involved in cytokine storm initiation and amplification, particularly in COVID-19 ARDS (22). Anakinra is an IL-1-receptor antagonist blocking the effect of both IL-1α and IL-1β. IL-1β leads to the production of IL-6 (23). However, tocilizumab blocks IL-6-mediated signal transduction by targeting both the membrane and soluble forms of the IL-6 receptor (24). Thus, it seems logical that the use of tocilizumab or anakinra could be effective in COVID-19. A large amount of data in the literature confirm the efficacy of the two anti-IL drugs (10, 25–27).

However, to date, randomized trials comparing the efficacy of different agents are lacking. The effect of tocilizumab and anakinra seems similar among all the meta-analyses (28–31). One Turkish study compared many patients receiving tocilizumab or anakinra using propensity score matching (15). The authors concluded a lower mortality and ICU transfer rate in the anakinra group. However, in this cohort, patients received very high doses of anakinra (600 mg daily mean versus 100 mg daily in most studies) and were also treated with hydroxychloroquine and favipiravir. Finally, the patients in the tocilizumab group received less corticosteroid than in the anakinra group, which is a major bias. Langer-Gould et al. (32) compared 52 patients treated with tocilizumab to 41 patients treated with anakinra using a propensity score matching and found no statistical difference in mortality. Some other studies, with a few participants, have directly compared tocilizumab to anakinra. Iglesias-Julian et al. (33) showed the same mortality under tocilizumab or anakinra. Patoulias et al. (14) performed a meta-analysis on three non-randomized studies comparing 125 patients under tocilizumab to 112 patients under anakinra and found lower mortality in the anakinra group. Unfortunately, they did not adjust their results with confounding factors. To the best of our knowledge, here, we report one of the largest cohorts of patients treated with tocilizumab compared to anakinra using an adjustment on confounding factors.

We acknowledge some limitations of this study. First, it was retrospective, but we could compare the patients using propensity score matching despite the absence of randomization. We used a combination of clinical, biological, and radiological prognosis factors to build our propensity score matching. Second, the patients were recruited over a large period, including patients infected by different variants of SARS-CoV-2. We could not add variants in the propensity score matching because there were too much missing data, which would have led to the inability to build the propensity score matching. Furthermore, we included a few patients with the omicron variant, which is now dominating. However, there is no evidence that the SARS-CoV-2 omicron variant changes the response to the anti-inflammatory therapy (34, 35). In effect, even if the omicron variant is less severe than the others, thanks to the propensity score matching, we could compare patients with COVID-19 with the same severity (according to the extent of lung involvement, the CRP level, or the rate of high-flow requirement before anti-IL introduction, which are well-known factors of severity). We also acknowledge the lack of assessment of inflammatory biomarkers such as interleukin-6 and soluble urokinase plasminogen activator receptor (suPAR) at baseline. Nevertheless, their utility was not clear, hyperinflammation was defined by CRP and ferritin levels, and IL-6 and suPAR are not routinely available biomarkers in all the centers. Furthermore, the two groups could not be matched on anti-spike monoclonal antibodies (using the optimal method) because no patients in the tocilizumab group have received these treatments (they were not available during the second and third waves when most patients receiving tocilizumab were included). Therefore, we performed a second propensity score matching, in which the use of anti-spike monoclonal antibodies was balanced between the two groups. Despite the small sample size, similar results were found with this method. Moreover, the rate of antibiotic prescriptions was different between the two groups. However, there is no evidence in the literature showing that antibiotics influence the response to anti-IL drugs or the evolution of COVID-19 (36–38).

Since the efficacy of tocilizumab seems to be similar to the efficacy of anakinra, clinicians should consider the cost of the treatment to choose it. In France, the cost of a 10-day anakinra course is lower than that of an 8 mg/kg dose of tocilizumab. In our study, the average cost of tocilizumab per patient was 1,052€/1,124 USD compared to 189€/202 USD per patient treated with anakinra. Moreover, the half-life of anakinra [a few hours (39)] is lower than that of tocilizumab [14–21 days (40)]. In patients with secondary infection due to the anti-IL drug, the infection would be easier to manage with a short half-life therapy such as anakinra than with tocilizumab.

Despite the small sample sizes of groups after propensity score matching, our study showed the same efficacy and secondary infection risk of tocilizumab and anakinra to treat severe COVID-19. Thus, anakinra and tocilizumab represented equivalent therapies in conjunction with corticosteroids. Our results need to be confirmed in larger randomized studies in order to choose the most effective and personalized treatment plans for each patient.

## Data availability statement

The raw data supporting the conclusions of this article will be made available by the corresponding authors, upon reasonable request.

## Ethics statement

The studies involving human participants were reviewed and approved by Portail d’Accès aux Données de Santé - Assistance Publique Hôpitaux de Marseille. Written informed consent for participation was not required for this study in accordance with the national legislation and the institutional requirements.

## Author contributions

RA, FC, PS, PV and AD contributed to the design of the study. All the authors participated in the enrolment of study participants and data collection. RA and PS analyzed the data. RA, FC, PV and AD drafted the manuscript. All authors contributed to the data interpretation and in revising the final manuscript. All authors contributed to the article and approved the submitted version.
